# Nutrition and Physical Activity in Child Care Centers: the Impact of a Wellness Policy Initiative on Environment and Policy Assessment and Observation Outcomes, 2011

**DOI:** 10.5888/pcd10.120232

**Published:** 2013-05-23

**Authors:** Rodney Lyn, Joyce Maalouf, Sarah Evers, Justin Davis, Monica Griffin

**Affiliations:** Author Affiliations: Joyce Maalouf, Sarah Evers, Justin Davis, Georgia State University, Atlanta, Georgia; Monica Griffin, Georgia Department of Early Care and Learning, Atlanta, Georgia.

## Abstract

**Introduction:**

The child care environment has emerged as an ideal setting in which to implement policies that promote healthy body weight of children. The purpose of this study was to assess the effect of a wellness policy and training program on the physical activity and nutrition environment in 24 child care centers in Georgia.

**Methods:**

We used the Environment and Policy Assessment and Observation instrument to identify changes to foods served, staff behaviors, and physical activity opportunities. Observations were performed over 1 day, beginning with breakfast and concluding when the program ended for the day. Observations were conducted from February 2010 through April 2011 for a total of 2 observations in each center. Changes to nutrition and physical activity in centers were assessed on the basis of changes in scores related to the physical activity and nutrition environment documented in the observations. Paired *t* test analyses were performed to determine significance of changes.

**Results:**

Significant improvements to total nutrition (*P* < .001) and physical activity scores (*P* < .001) were observed. Results indicate that centers significantly improved the physical activity environments of centers by enhancing active play (*P* = .02), the sedentary environment (*P* = .005), the portable environment (*P* = .002), staff behavior (*P* = .004), and physical activity training and education (*P* < .001). Significant improvements were found for the nutrition environment (*P* < .001), and nutrition training and education (*P* < .001).

**Conclusion:**

Findings from this study suggest that implementing wellness policies and training caregivers in best practices for physical activity and nutrition can promote healthy weight for young children in child care settings.

## Introduction

An estimated 12 million children aged 5 years or younger spend 22.5 hours per week in child care centers where they consume most of their daily calories (1,2). In the context of the national problem of childhood obesity and its growing prevalence in young children, experts have identified child care centers as an ideal setting for early intervention in childhood obesity (3–5). Childhood obesity is a consequence of environments that disrupt the balance of energy intake and energy expenditure (6). Obesogenic environments consist of social norms and environmental factors that facilitate unhealthy behaviors around diet and physical activity (7). The healthy weight environment of child care centers is influenced by the quality of foods served, the time children spend being physically active, caregiver behaviors, and the presence of nutrition and physical activity wellness policies (5).

Research shows that children in child care do not meet 2010 Dietary Guidelines for Americans *recommendations* and that few structured opportunities exist for physical activity (8–10). Less is known about how caregiver interactions with children affect eating and physical activity behaviors of children (11,12). Small and consistent changes to school environments can mitigate the prevalence of overweight in youth (13,14); however, more research is needed to provide evidence of effective approaches to improving nutrition and physical activity practices in child care facilities.

Reviews of interventions to prevent childhood obesity in child care settings demonstrate a rapidly growing research area (5,15). Most evaluated interventions are designed to affect the nutritional quality of foods, intensity of and opportunities for physical activity, and interactions of caregivers with children (5). A small number of studies have been conducted to improve child care nutrition and physical activity environments (2,16–18); to our knowledge, no programs have been evaluated previously that use local wellness policies to improve child care environments. The purpose of this study was to assess the effect of a wellness policy and training program on the physical activity and nutrition environment in 24 child care centers in Georgia.

## Methods

### Program rationale and design

The Child Nutrition and Women, Infants, and Children Reauthorization Act of 2004 included a provision requiring local education agencies participating in national nutrition programs (ie, National School Lunch Program, School Breakfast Program, and Special Milk Program) to adopt and implement local wellness policies in schools (19).The goal of the mandate was to promote wellness and health by focusing on nutrition education, physical activity, and other school-based activities to create healthy school environments. Story et al (14) previously recommended that child care centers develop written policies that comply with the Dietary Guidelines for Americans and ensure appropriate levels of and types of physical activity (10). Officials from Georgia’s Department of Early Care and Learning (DECAL) saw value in applying the mandate in child care facilities (20).

From February 2010 through April 2011, a program was conducted in southwest Georgia that focused on training caregivers in adoption and implementation of 6 wellness policies designed to improve nutrition and physical activity practices. The purpose of this study was to assess the effect of the program on the physical activity and nutrition environment in child care centers. A US Department of Agriculture (USDA) Team Nutrition Grant supported the program, Caregivers Promoting Healthy Habits, and provided funding for up to 30 centers. The program sought to provide evidence that wellness policy training and support to child care centers statewide in subsequent years would lower childhood obesity rates.

Centers selected and adopted 6 of 12 model policies (Table 1). Centers were provided technical assistance by a registered dietitian and up to $2,000 to support implementation. An expert on physical activity provided input on the development, implementation, and evaluation of program activities related to physical activity. Center directors and staff participated in 4 trainings on nutrition and physical activity, menu planning, food safety, and healthy habits consistent with the wellness policies. Trainings were conducted in group settings where participants could exchange ideas with other child care center staff. The program objectives were to 1) introduce child care providers to the concept and benefits of wellness policies; 2) help child care providers develop and implement wellness policies in their centers; 3) support centers through training, technical assistance, and funding; and 4) evaluate the program’s effect on the nutrition and physical activity environment of centers.

**Table 1:** Proposed Voluntary Wellness Policies and Percentage of Centers Selecting Policies, Wellness Policy Initiative on Environment and Policy Assessment and Observation (EPAO) Outcomes, Georgia, 2010–2011

Description of Wellness Policy and Activities (R = Required, E = Encouraged)^a^	n (%)^b^
**Policy 1. Breastfeeding is promoted and adequately supported.**	**1 (4)**
Provide refrigeration for storing expressed breast milk and feed this milk to the child as requested by the parent/guardian. (R)	
Use thawed breast milk within 24 h and fresh breast milk within 48 h. (R)	
**Policy 2. Foods served to children exceed the US Department of Agriculture Child and Adult Care Food Program guidelines and meet the Dietary Guidelines for Americans/MyPyramid for Preschoolers recommendations (** 10 **).**	**10 (42)**
Children in a part-day child care center receive meals/snacks that provide one-third of the child’s nutritional needs, while children in a full-day child care program must receive meals/snacks that provide half to two-thirds of the child’s nutritional needs. (R)	
Children are served foods and beverages that promote acceptance of a variety of foods. (R)	
Children are served fresh fruits and vegetables daily. (E)	
Children are offered healthful beverages such water; 100% juice with no added sugars, artificial sweeteners, flavoring, and colors; and low-fat or fat-free milk for children older than age 2. (R)	
Children are served new and familiar foods. (R)	
A dietitian is consulted to ensure that a variety of nutritious, appealing, and age-appropriate foods is served. (E)	
**Policy 3. Children always have access to safe drinking water and are encouraged to drink water frequently throughout the day.**	**15 (63)**
Adults model frequent drinking of water instead of drinking other fluids. (R)	
**Policy 4. The daily schedule promotes a relaxed and adequate period for meals and snacks.**	**9 (38)**
Quiet time precedes meals to promote relaxed eating. (R)	
Meal schedules are long enough to allow for conversation and for serving food to the children several times, if necessary. (R)	
**Policy 5. Food and physical activity are not used as incentives or punishment.**	**2 (8)**
Children are neither rewarded nor punished with physical activity. (R)	
**Policy 6. Children serve themselves during meals and snacks with adult supervision.**	** 11 (46)**
Food is served in a manner that allows children to select amounts and varieties of foods they will eat. (R)	
Food is served in a form that young children can eat without assistance, when appropriate. (R)	
Adults eat with children and model good eating habits by consuming only healthful foods and beverages in the presence of children. (R)	
**Policy 7. Nutrition and physical activity are taught as specific learning objectives and woven into activities throughout the day.**	**24 (100)**
A nutrition and/or physical activity curriculum is adopted. (E)	
Books relating to food, eating, and physical activity are read to children before or after meals and snacks. (E)	
Activities and games that increase knowledge and acceptance of a variety of foods and physical activities are planned. (R)	
Children are engaged in planning and preparing food when appropriate. (E)	
Educational tools are used to promote healthy eating and physical activity. (R)	
**Policy 8. Parents are partners in the task of fostering healthy eating and physical activity habits for children.**	**23 (96)**
Daily information is provided to parents about their child’s activities and needs including eating and physical activity. (R)	
Information and ideas are provided to families that discuss how they can support healthy nutrition and physical activity practices. (R)	
A written policy on nutrition, food service, and physical activities is shared with parents before a child enters child care. (R)	
Information is provided to parents about being healthy role models for their children. (E)	
**Policy 9. Sanitation, hygiene, and food handling are monitored to ensure a healthy environment.**	**7 (29)**
A policy is developed and shared with parents regarding food brought from home that addresses food safety and nutrition and requires prior approval of any foods brought for sharing. (R)	
Hand washing is stressed as the first defense against spreading germs. Adults and children wash their hands frequently. (R)	
When appropriate, sinks, soap, and paper towels are placed at children’s height so hand washing is easy and comfortable. (R)	
Adults are trained and monitored on procedures for preparing, serving, and storing food and on sanitizing and disinfecting dishes, equipment, and surfaces. US Department of Agriculture food sanitation standards are observed. (R)	
Toys and playground equipment are regularly cleaned. (R)	
**Policy 10. At least 60 minutes of physical activity are scheduled daily, and screen time is limited for toddlers and preschoolers.**	**7 (29)**
Physical activity is scheduled throughout the day as recommended by the National Association for Sport and Physical Education (31) in the physical activity guidelines for infants and toddlers and is a part of the regular schedule. (R)	
Unstructured playtime and planned movement experiences, both indoor and outdoor, are included in the schedule. (R)	
Center will limit television and video viewing to less than 1 hour per day, including educational programs, for children older than age 2. (R)	
Center will ensure that children younger than age 2 are not viewing television or videos. (R)	
**Policy 11. Physical activities, equipment, and facilities are developmentally appropriate and safe and meet the National Association for Sport and Physical Education (** 31 **) guidelines for young children.**	**11 (46)**
Activities focus on age-appropriate motor skills. Children have the opportunity to practice important skills. Cooperation is stressed while competition is avoided. (R)	
Equipment and facilities are routinely monitored for safety. (R)	
Activities and equipment are age-appropriate, and all children, regardless of age, have equipment to play on that provides them the chance to have fun and be active. (R)	
Staff participate in and model physical activities for the children and facilitate/encourage children’s movement and exploration of their environment. (R)	
**Policy 12. Staff is adequately trained about nutrition and physical activities for young children.**	**23 (96)**
Wellness information and activities for employees are provided. (R)	
Staff is provided with training about nutrition and physical activity for young children. (R)	

a Policies follow standards and practices advocated by US Department of Agriculture, American Academy of Pediatrics (32), National Association for Sport and Physical Education, and Centers for Disease Control and Prevention. Policies are based on best practices for nutrition and physical activity in early care settings developed by the state of Iowa and standards and indicators of quality identified by Georgia early childhood experts.

b Number and percentage of centers (of 24) that selected the specified policy.

### Setting

The southwest region of Georgia was selected for program implementation because many of the counties ranked low on health status indicators including child poverty, adult obesity, and high school graduation rates. In December 2010, DECAL issued an abbreviated Request for Application to centers, held a pre-application conference to introduce centers to the program, and distributed previously developed nutrition and physical activity materials and curricula. Interested centers were asked to respond to the request by submitting a self-assessment to identify areas related to nutrition and physical activity that needed improvement, 6 self-identified wellness policies, and proposed activities related to implementation of policies. At baseline, February 2010, 24 child care centers were enrolled in the program.

Participation criteria for the program included that centers were licensed by the state and were not considered a child care or prekindergarten program in an elementary school. All centers were required to be enrolled in the Child and Adult Care Food Program (CACFP), a USDA program that provides reimbursement for nutritious meals and snacks in eligible child and adult care centers. For-profit centers (n = 14) and nonprofit centers (n = 10) were included in the program; 4 centers offered the Head Start program and only 1 center was accredited by the National Association for the Education of Young Children. The centers served a total of 2,042 children aged 2 to 5 years (range, 40–245 children per center) during the program. All centers provided full-day programs. The Georgia State University institutional review board approved the protocol. Directors, representing child care centers, provided written informed consent.

### Evaluation measures

The study used a pretest-posttest design to assess the child care environment by using the Environment and Policy Assessment and Observation (EPAO) instrument (21). The EPAO is validated for assessing the nutrition and physical activity environment in child care centers; at the time of the study, it was the only validated instrument available. The EPAO was developed to evaluate the Nutrition and Physical Activity Self-Assessment for Child Care (NAP SACC) intervention (2).

Baseline observations were conducted in 24 centers, and final observations made in 22 centers; 2 centers dropped out of the study because of center closings. Center visits were unannounced, and observations were performed by 1 registered dietitian trained to conduct the EPAO. The EPAO protocol consists of 1 full-day visit to each center and includes direct observation of the nutrition and physical environment and a documented review of activities. The observer randomly selected 1 classroom of children aged 2 to 5 years for the observation. During naptime, the observer evaluated other portions of the facility outside of the classroom, assessing the presence of vending machines, the type of kitchen used, the characteristics of the outdoor play environment, posted policies and wellness information, and other center characteristics. The observer reviewed lesson plans, fundraising documents, menus, staff handbooks, and nutrition and physical activity policies and curricula. Observations began at breakfast and lasted until the conclusion of the child care program (ie, after-school care programs were not observed).

### Statistical analysis

EPAO data were converted to a numerical scale and recorded with a scoring guideline that grouped data into subscales (Table 2). Scores were averaged within subscales and multiplied by 10, yielding a final score (of 20 possible points) for nutrition and physical activity environments. Higher scores on the observational assessment represented a healthier physical activity or nutrition environment at baseline and final. Means and standard deviations were calculated to describe the sample and group differences. Baseline and final scores were compared by using paired *t* tests. All analyses were performed using PASW Statistics 18 (IBM, Chicago, Illinois). *P* values at or greater than .05 were considered significant.

**Table 2:** Nutrition and Physical Activity Environment Subscale Items Scored, Wellness Policy Initiative on Environment and Policy Assessment and Observation (EPAO) Outcomes, Georgia, 2010–2011

Category	Content Summary
Nutrition factors scored	
Fruits and vegetables	Types and frequency of fruits and vegetables served; use of butter, margarine, or meat fat on vegetables; added fat in vegetables
Grains/low fat meats	Lean meats/fish; beans/lentils; high fiber and whole grains served
High sugar/high fat foods	Fried vegetables and meats; high fat meats; high sugar and high fat foods and condiments served
Beverage	Servings of 100% fruit juice; availability and encouragement of drinking water, servings of sugary drinks, types and servings of milk
Staff behavior	Staff do not push children to eat more than desired, food not used to control behavior; staff encourage children to try new foods, staff sit with children during lunch, staff eat the same foods as children
Nutrition environment	No soda and vending machines, family-style meals served; display of posters, pictures, or books about nutrition
Nutrition training and education	Nutrition education for children with documented curriculum; staff communicate with children about healthy foods; nutrition training for staff
Nutrition policy	Written guidelines address holiday/celebration foods, review of fundraising projects for healthy foods if food-based, written policy on nutrition and food service evident
**Physical activity factors scored**	
Active play	Frequency and length of active and structured play, outdoor active play
Sedentary behavior	Children not seated for more than 30 minutes at a time, TV viewing observed (minutes); videogame playing observed (minutes)
Sedentary environment	No TV or computer in room for use by children; posters, pictures, or books about physical activity available
Portable environment	Variety of mobile physical activity equipment (balls, jump ropes, riding toys, etc.) available
Fixed environment	Fixed play items (basketball hoop, swing, running space, etc.) available
Staff behavior	Staff do not restrict active play as punishment; staff join in active play; staff make positive statements about physical activity to children
Physical activity training and education	Physical activity education for children with documented curriculum; extracurricular programs; physical activity training for staff; documentation of physical activity education/workshop materials
Physical activity policy	Center has written policy on physical activity

## Results

At baseline, the average EPAO nutrition and physical activity total scores were 12.1 (standard deviation [SD] 1.8) and 10.8 (SD 1.3) (Table 3). Mean nutrition subscale scores ranged from 8.4 (nutrition training and education) to 15.4 (staff behavior). Mean physical activity subscale scores ranged from 5.5 (physical activity training and education) to 16.8 (physical activity policy score). When we compared baseline scores with posttest scores, we found significant improvements to both total nutrition and physical activity scores with mean differences of 1.9 (*P* < .001) for nutrition and 2.8 (*P* < .001) for physical activity.

**Table 3:** Baseline and 12-Month Scores, Environment and Policy Assessment and Observation (EPAO) in 22 Child Care Centers, Georgia, 2010–2011

Subscale Item	Baseline Mean (SD)	12-Month Mean (SD)	Mean Difference^a^	P Value^b^
**Nutrition EPAO**				
Fruits and vegetables	11.8 (2.7)	12.0 (1.7)	0.2	.79
Grains/low fat meats	11.0 (3.8)	9.9 (3.5)	−1.1	.25
High sugar/high fat foods	12.0 (2.4)	13.0 (1.6)	1.0	.06
Beverage	12.9 (2.6)	13.5 (2.0)	0.6	.36
Staff behavior	15.4 (3.4)	15.9 (4.0)	0.5	.64
Nutrition environment	12.7 (4.1)	16.4 (4.9)	3.6	<.001
Nutrition training and education	8.4 (4.6)	16.9 (3.0)	8.5	<.001
Nutrition policy	12.7 (2.0)	14.9 (3.7)	2.1	.05
**Total nutrition score**	**12.1 (1.8)**	**14.0 (1.2)**	**1.9**	**<.001**
**Physical activity EPAO**				
Active play	8.9 (3.5)	11.1 (4.5)	2.1	.02
Sedentary behavior	11.2 (4.6)	11.8 (4.6)	0.6	.56
Sedentary environment	10.0 (5.7)	13.0 (5.6)	3.0	.005
Portable environment	9.9 (4.6)	13.9 (3.5)	4.0	.002
Fixed environment	12.8 (2.6)	12.3 (2.5)	−0.5	.31
Staff behavior	11.3 (5.2)	15.3 (5.1)	4.0	.004
Physical activity training and education	5.5 (4.3)	14.3 (4.4)	8.9	<.001
Physical activity policy	16.8 (4.8)	16.8 (4.8)	0	NA^c^
**Total physical activity score**	**10.8 (1.3)**	**13.6 (1.7)**	**2.8**	**<.001**

Abbreviation: NA, not applicable.

a Mean difference between baseline and 12-month scores.

b Paired *t* test used to test mean differences based on baseline and 12-month means for all centers; *P* ≤ .05 considered significant.

c No change occurred for the subscale “physical activity policy” as it related to the EPAO standards for physical activity policy.

Significant improvements from baseline to final observations were found for the nutrition environment means (difference: 3.6, *P* < .001). Nutrition training and education scores also improved significantly (difference: 8.5, *P* < .001). Physical activity subscales showed significant improvement from baseline to final observations of active play (difference: 2.1, *P* = .02), sedentary environment (difference: 3.0, *P* = .005), and portable environment (difference: 4.0, *P* = .002). The score addressing staff behavior and physical activity improved significantly (difference: 4.0, *P* = .004), as did physical activity training and education (difference: 8.9, *P* < .001).

## Discussion

Results suggest that adoption of wellness policies can improve the nutrition and physical activity environment in child care centers. This intervention focused on the adoption of wellness policies, increasing staff knowledge of physical activity and nutrition best practices through trainings, and guidance on the implementation of policies. Participating centers improved significantly on a range of EPAO domains: the physical activity environment, active play, sedentary environment, portable environment, staff behavior, and physical activity training and education. We also found significant improvements to the nutrition environment, nutrition training and education, and nutrition policy.

At baseline, most centers had a physical activity policy in place, which may explain why changes to the physical activity policy score were not observed. Results suggest, however, that having a physical activity policy may not correspond with actual physical activity practices; centers had areas with room for improvement, particularly the active play of children. A study that reviewed policies related to active playtime in child care centers found that although 48% of centers had active play policies, language was vague and included phrases such as “children go outside daily, weather permitting” (22). Researchers have suggested that children at preschool age should engage in 120 minutes of physical activity each day including at least 2 teacher-led activities and 2 outdoor activities (22,23). Our study suggests that the adoption of wellness policies and delivery of trainings focused on improving physical activity may increase structured and outdoor playtime.

The portable physical activity environment also improved significantly among participating centers. Centers improved physical activity environments by acquiring new equipment or making use of existing equipment. Portable play equipment, including items such as riding and push/pull toys, jumping toys, ball play, and floor play, has been shown to encourage children’s physical activity (18,24). Results from a study in 20 child care centers found that the availability of portable play equipment influenced physical activity more than availability of fixed equipment (24). Despite overall improvements to this score, the nature of changes to portable equipment used is unclear. Research is needed to identify the optimal combination of fixed and portable play items as well as how these play materials should be incorporated into playtimes to best promote physical activity.

Centers showed significant improvement in the behavior score related to staff joining in active play, making positive statements about physical activity to children, and not restricting activity as punishment. Together with research that demonstrates the impact of supportive staff behaviors on child physical activity and dietary behaviors, these results suggest that the adoption of wellness policies and delivery of staff trainings increased caregivers’ ability to participate in promoting healthy child behaviors (4,22).

The overall nutrition score for participants reflected significant improvements, yet there were fewer EPAO nutrition domains that significantly changed than physical activity EPAO domains. Centers continue to have an opportunity to improve healthy eating for children, particularly the servings of fresh fruits, vegetables, and whole grains. At baseline and at 12 months, the average fruits and vegetables and the average grains/low fat meals scores were low. Findings from this study are consistent with others that found that child care centers offered menus low in fresh fruits and vegetables and whole grains (9,25,26).

Although we found significant improvements in the nutrition environment, nutrition training and education, and nutrition policy, other scales did not improve. Centers in this study could enhance their overall mealtime and feeding environments to support healthy eating for young children (25,27). Although data linking training and education to improved health outcomes in child care settings are limited, education opportunities have the potential to help promote healthy eating in child care settings (25). Behaviors among caregivers are important for facilitating improvements to the nutritional environment (27,28). Specifically, caregivers affect children’s eating habits through role-modeling, and caregivers’ participation in mealtimes can encourage children to try new foods (11). In particular, providers could improve behavior scores by enhancing interactions with children related to hunger and satiety (27–29). All centers in this study were CACFP-funded. Other research has suggested that CACFP-participating centers have consistently more supportive feeding environments than nonparticipating centers (30), which may explain why supportive staff behaviors around nutrition did not change.

Overall, centers improved their total scores for nutrition and physical activity. The results of this study are strengthened because this is the first evaluation of a childhood obesity prevention program in child care centers in the state of Georgia. This study is one of few interventions designed to affect the child care environment by using local wellness policies related to nutrition and physical activity. The dropout rate of this program was very low (only 2 centers, 8%). We used a validated instrument and data collection was performed by 1 registered dietitian to ensure standardization of observational data.

Our study had limitations. The study lacked a large sample size and a methodology to objectively assess changes in children’s dietary intake and physical activity levels. Our ability to evaluate the direct effect of wellness policy implementation on child nutrition and physical activity was limited. Future studies should use a comparison group of centers to objectively evaluate this program while incorporating child-level health outcomes.

The child care setting provides a host of opportunities to improve the nutrition and physical activity environment. The findings of this study suggest that wellness policies implemented by child care center staff may contribute to a healthier child care environment. This study adds to the limited research base on wellness policies and the nutritional and physical activity environments in child care centers.
